# Clinical correlates of social cognition after an ischemic stroke: preliminary findings

**DOI:** 10.1590/1980-57642021dn15-020010

**Published:** 2021

**Authors:** Maria de Fátima Dias de Souza, Maíra Glória de Freitas Cardoso, Érica Leandro Marciano Vieira, Natália Pessoa Rocha, Talita Hélen Ferreira e Vieira, Alberlúcio Esquirio Pessoa, Vinicius Sousa Pietra Pedroso, Milene Alvarenga Rachid, Leonardo Cruz de Souza, Antônio Lúcio Teixeira, Aline Mansueto Mourão, Aline Silva de Miranda

**Affiliations:** 1 Laboratório Interdisciplinar de Investigação Médica, Faculdade de Medicina, Universidade Federal de Minas Gerais - Belo Horizonte, MG, Brazil.; 2 The Mitchell Center for Alzheimer’s Disease and Related Brain Disorders, Department of Neurology, McGovern Medical School, The University of Texas Health Science Center at Houston - Houston, TX, USA.; 3 Departamento de Morfologia, Instituto de Ciências Biológicas, Universidade Federal de Minas Gerais - Belo Horizonte, MG, Brazil.; 4 Departamento de Fisioterapia, Faculdade Sete Lagoas - Sete Lagoas, MG, Brazil.; 5 Hospital Metropolitano Odilon Behrens - Belo Horizonte, MG, Brazil.; 6 Departamento de Patologia, Instituto de Ciências Biológicas, Universidade Federal de Minas Gerais - Belo Horizonte, MG, Brazil.; 7 Santa Casa BH Ensino e Pesquisa - Belo Horizonte, MG, Brazil.; 8 Neuropsychiatry Program, Department of Psychiatry & Behavioral Sciences, McGovern Medical School, University of Texas Health Science Center at Houston - Houston, TX, USA.; 9 Departamento de Fonoaudiologia, Faculdade de Medicina, Universidade Federal de Minas Gerais - Belo Horizonte, MG, Brazil.

**Keywords:** cognition, cognitive dysfunction, neurobehavioral manifestations, depression, stroke, cognição, disfunção cognitiva, manifestações neurocomportamentais, depressão, acidente vascular cerebral

## Abstract

The co-occurrence of post-stroke behavioral disorders and cognitive impairment has been extensively investigated. However, studies usually do not include social cognition among the assessed cognitive domains.

**Objective:**

To investigate the potential association between facial emotion recognition, a measure of social cognition, and behavioral and cognitive symptoms in the subacute phase of ischemic stroke.

**Methods:**

Patients admitted to a Stroke Unit with ischemic stroke were followed up to 60 days. At this time point, they were evaluated with the following tools: Mini-Mental State Examination (MMSE); Frontal Assessment Battery (FAB); Visual Memory Test of the Brief Cognitive Battery (VMT); Phonemic Verbal Fluency (F-A-S Test); Digit Span; Facial Emotion Recognition Test (FERT) and Hospital Anxiety and Depression Scale (HADS). A control group composed of 21 healthy individuals also underwent the same evaluation.

**Results:**

Eighteen patients with ischemic stroke were enrolled in this study. They had similar age, sex and schooling years compared to controls. Depression symptoms and episodic memory deficits were significantly more frequent in patients compared to controls. The recognition of sadness expression positively correlated with the levels of anxiety and depression, while and the recognition of fear expression negatively correlated with depression in the stroke group.

**Conclusions:**

After an ischemic stroke, patients exhibit impairment in social cognition skills, specifically facial emotion recognition, in association with behavioral symptoms.

## RESUMO.

A co-ocorrência de distúrbios comportamentais e comprometimento cognitivo pós-acidente vascular cerebral (AVC) é amplamente descrita na literatura. No entanto, os estudos geralmente não incluem a cognição social entre os domínios cognitivos avaliados.

**Objetivo:**

Investigar a potencial associação entre o reconhecimento da emoção facial, uma medida da cognição social, e os sintomas comportamentais e cognitivos na fase subaguda do AVC isquêmico.

**Métodos:**

Pacientes internados em uma Unidade de AVC com AVC isquêmico foram acompanhados até 60 dias, quando foram avaliados com os seguintes instrumentos: Mini-Exame do Estado Mental (MEEM); Bateria de Avaliação Frontal (FAB); Teste de Memória Visual da Bateria Cognitiva Breve (VMT); Fluência Verbal Fonêmica (Teste F-A-S); Span de dígitos; Teste de Reconhecimento de Emoção Facial (FERT) e Escala Hospitalar de Ansiedade e Depressão (HADS). Um grupo controle constituído por 21 indivíduos saudáveis também foi submetido à mesma avaliação.

**Resultados:**

Dezoito pacientes com AVC isquêmico foram incluídos no presente estudo, apresentando idade, sexo e anos de escolaridade semelhantes aos do grupo controle. Os sintomas de depressão e déficits de memória episódica foram significativamente mais frequentes em pacientes com AVC. O reconhecimento da expressão de tristeza correlacionou-se positivamente com os níveis de ansiedade e depressão, ao passo que o reconhecimento da expressão de medo correlacionou-se negativamente com depressão no grupo de AVC.

**Conclusões:**

Após um AVC isquêmico, pacientes podem apresentar alterações de cognição social, especificamente de reconhecimento da emoção facial, em associação com sintomas comportamentais.

## INTRODUCTION

Stroke is a common, serious, and disabling global health problem. The development of post-stroke behavioral disorders and cognitive impairment leads to worse clinical prognosis.^1^ Although these disorders have been extensively described following cerebral ischemic events, these studies usually do not include social cognition among the assessed cognitive domains.^1^ ^,^ ^2^ ^,^ ^3^ ^,^ ^4^ ^,^ ^5^ ^,^ ^6^ ^,^ ^7^ ^,^ ^8^ ^,^ ^9^ ^,^ ^10^

Social cognition refers to cognitive processes related to perception and interpretation of the social environment.^11^ For instance, the ability to recognize other person’s emotions from his/her facial expression is essential for human interactions.^12^ ^,^ ^13^ Over the last two decades, the study of social cognition has gained greater attention in the literature. However, only a few studies evaluated facial emotion recognition after stroke, reporting decreased ability to recognize emotions in patients when compared with healthy controls.^14^ ^,^ ^15^ ^,^ ^16^ ^,^ ^17^ Moreover, these studies did not investigate potential interactions among behavior, general cognition and social cognition measures.

Identifying potential interactions among these discrete domains is a promising area of investigation, especially given the role played by these factors on rehabilitation and, hence, prognosis of patients with stroke.

Stroke significantly affects the life of the patient on multiple levels, including physical, cognitive, behavioral, and social ones.^14^ The current study focused on the evaluation of facial emotion recognition, a marker of social cognition, investigating whether it would be associated with a series of cognitive domains, as well as depression and anxiety symptoms in the subacute phase of ischemic stroke.

## METHODS

### Participants

This study is part of a research project approved by the Research Ethics Committee of the Universidade Federal de Minas Gerais (Project, CAAE: 02811212.5.3001.5129) and by the Research Ethics Committee of the Hospital Municipal Odilon Behrens (Number 2.515.900, February 27, 2018). Here, we present cross-sectional data on clinical assessments of behavior, cognition and social cognition (i.e., facial emotion recognition) of the study participants.

Eighteen patients who were followed up to 60 days after their stroke were enrolled in this study. Inclusion criteria were patients over 18 years old with the diagnosis of ischemic stroke. The exclusion criteria were: comorbidity with other neurological diseases (traumatic brain injury, epilepsy and other); neurodegenerative diseases; psychiatric disorders (schizophrenia, bipolar disorder, major depression, generalized anxiety and panic disorders); inflammatory (i.e. myelitis, sarcoidosis) and infectious diseases (HIV, neurosyphilis and others). Patients with altered level of consciousness according to the Glasgow Coma Scale (≤14) and aphasia according to the National Institutes of Health Stroke Scale (NIHSS) at admission were also excluded.

**Figure 1.**
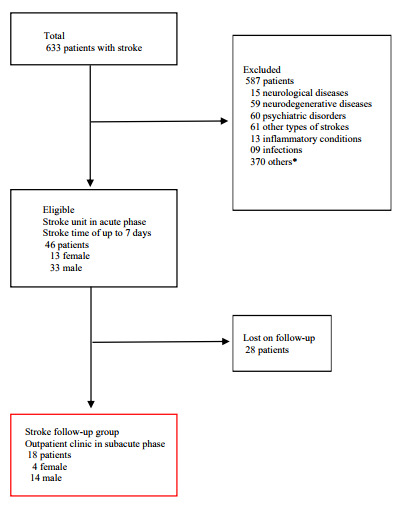
Flow diagram of patient recruitment.

Healthy participants with comparable age, sex, and educational level were recruited from the local community (control group). This group comprised people with no previous diagnosis of stroke or any other neurological, neurodegenerative, psychiatric, inflammatory or infectious diseases. The recruitment process and study design are outlined in Figure 1.

*Ictus>7 days; Glasgow Coma Scale≤14; cancer, chronic obstructive pulmonary disease; postoperative.

### Clinical assessment

The neuropsychological assessment of the stroke group was performed during an outpatient clinic visit between 45 and 60 days after the ictus. The following tools were applied: (i) the Mini-Mental State Examination (MMSE), as a measure of general cognition;^18^ ^,^ ^19^ (ii) the Frontal Assessment Battery (FAB) for the evaluation of executive functions, including six subtests: similarities, lexical fluency (letter S), motor series, conflicting instructions, go/no-go and prehension behavior;^20^ (iii) the Visual Memory Test of the Brief Cognitive Battery (VMT)^21^ ^,^ ^22^ that assesses incidental memory, immediate memory, learning, late recall and recognition; (iv) the Phonemic Verbal Fluency (F-A-S Test);^23^ (v) the Digit Span for attention and working memory;^24^ and (vi) the Facial Emotion Recognition Test (FERT) for social cognition.^11^ ^,^ ^25^

Besides cognitive tests, the Hospital Anxiety and Depression Scale (HADS) was used to detect symptoms of anxiety and depression.^26^ Neurological impairment was determined by the National Institutes of Health Stroke Scale (NIHSS) in the acute phase of the stroke.^27^

The same cognitive and behavioral battery was applied to controls.

### Statistical analysis

Categorical variables were expressed as frequencies and percentages. A bilateral p value lower than 0.05 was adopted as the level of significance for all statistical tests.

All variables were assessed for normality using the Shapiro-Wilk test, presenting a non-parametric distribution. Differences of sex and education between groups were tested by Pearson chi-square test. Differences of age and neuropsychological performance between groups were tested using the Mann-Whitney test.

A binary logistic regression was performed to determine which variables were significantly associated with stroke. A backward stepwise regression was used, and the following variables were included in the initial model: age, sex, FERT total score, HADS anxiety, HADS depression and VMT late recall. The backward stepwise selection was automatically performed using the SPSS software version 26.0 (SPSS Inc., Chicago, IL, USA), and the removal testing was based on the probability of the likelihood-ratio statistic based on conditional parameter estimates. The goodness of fit of the logistic regression model was assessed by the Hosmer-Lemeshow test, as well as a Receiver Operating Characteristic (ROC) curve.

To identify individuals with suggestive clinical anxious and depressive scores on HADS, a clinical cutoff score of 6 was used for HAD-D^26^ and of 7 for HAD-A.^28^ The Fisher’s exact test was applied to investigate differences in prevalence of anxiety and depression between groups.

## RESULTS

Table 1 shows sociodemographic characteristics and neuropsychological performance of stroke and control groups. Patients and controls have similar age, sex and educational level. Post-stroke patients had worse depressive symptoms and worse cognitive performance in cognitive domains than controls.

**Table 1.** Sociodemographic characteristic and neuropsychological performance of stroke follow-up and control groups.

		Control n=21 n (%) median (percentile 25-75)	Post-stroke n=18 n (%) median (percentile 25-75)	p-value
**Sociodemographic characteristic**				
Gender	Male	13 (62%)	14 (78%)	0.284*
	Female	8 (38%)	4 (22%)	
Education	Illiterate	1 (5%)	1 (6%)	0.921*
	1-4 years of schooling	5 (24%)	3 (17%)	
	5-8 years of schooling	7 (33%)	6 (33%)	
	9-10 years of schooling	0 (0 %)	1 (5%)	
	11 years of schooling	7 (33%)	6 (33%)	
	More than 11 years of schooling	1 (5%)	1 (6%)	
Age (years)	Mean±DPM	63.43±9.89	62.89±11.74	0.799^+^
	(Minimum-maximum)	(39-80)	(33-84)	
**Behavior, cognitive and social cognition tests**				
Anxiety	HADS_A	3 (2.50-5.00)	8 (2.00-10.00)	0.057 ^+^
Depression	HADS_D	2 (0.00-4.00)	5 (1.75-7.50)	**0.031** ^+^
Cognitive deficits	MEEM	28 (26.00-29.00)	29 (27.75-30.00)	0.161^+^
Executive functions	FAB	17 (16.00-17.00)	17 (14.75-17.25)	0.712^+^
Visual memory test	VMT_incidental_memory	8 (7.50-9.50)	8 (5.75-9.00)	0.101^+^
	VMT_immediate_memory	10 (9.00-10.00)	8 (7.00-9.00)	**0.002** ^+^
	VMT_learning	10 (9.00-10.00)	9 (8.00-10.00)	0.084^+^
	VMT_late recall	10 (8.50-10.00)	8.5 (8.00-9.25)	**0.044** ^+^
	VMT_recognition	10 (10.00-10.00)	10 (9.75-10.00)	**0.025** ^+^
Phonemic verbal fluency	FAS_animals	14.5 (12.25-16.75)	14 (11.00-15.25)	0.527^+^
	FAS_F	8 (4.50-11.00)	7.5 (4.75-11.00)	0.821^+^
	FAS_A	9 (6.50-12.00)	8.5 (5.00-10.00)	0.343^+^
	FAS_S	10 (6.00-12.00)	8.5 (5.00-13.00)	0.799^+^
Attention and working memory	DS_order_direct_right	8 (6.00-9.00)	9 (7.75-9.25)	0.209^+^
	DS_order_direct_span	5 (4.00-6.00)	6 (5.00-6.00)	0.187^+^
	DS_order_reverse_acertos	3 (2.00-4.00)	4 (2.00-5.00)	0.283^+^
	DS_order_reverse_span	3 (2.00-3.00)	3 (2.00-4.00)	0.279^+^
Social cognition	FERT	24 (21.50-27.50)	24.5 (22.00-27.25)	0.745^+^

n: number of patients; DPM: Average Standard Deviation; MEEM: Mini-Mental State Examination; FAB: Frontal Assessment Battery; HADS: Hospital Anxiety and Depression Scale; HADS_A: Anxiety subscale; HADS_D: Depression subscale; VMT: Visual Memory Test of the Brief Cognitive Battery; VMT_incidental_memory: VMT incidental memory; VMT_immediate_memory: VMT immediate memory; VMT_learning: VMT learning; VMT_late recall: VMT late recall; VMT_recognition: VMT recognition; FAS: Verbal Fluency Task; FAS_ animals: FAS semantic category for animals; FAS_F: FAS production of words beginning with F; FAS_A: FAS production of words beginning with A; FAS_S: FAS production of words beginning with S; FERT: Facial Emotion Recognition Test; *Pearson's chi-square test; ^+^Mann-Whitney test.

The mean NIHSS of the stroke group was 3.56±2.00, without any correlation with behavior, cognitive and social cognition tests.

Although there was no significant difference between patients and controls in FERT scores, we observed that among patients the recognition of sadness expression positively correlated with the levels of anxiety (Spearman’s rank correlation coefficient [rho]=0.587, p<0.05) and depression (rho=0.598, p<0.01). Conversely, the recognition of fear expression negatively correlated with depression symptoms in the stroke group (rho=0.481, p<0.05) (Table 2). In addition, VMT recall positively correlated with FERT neutral (rho=0.523, p=0.026) and FERT total scores (rho=0.478, p<0.05), and VMT recognition positively correlated with FERT total score (rho=0.554, p=0.017). These correlations were not found in controls.

**Table 2.** Spearman correlation between Facial Emotion Recognition Test with cognitive and behavioral tests at stroke follow-up group (n=18).

FERT	HADS anxiety	HADS depression	MMSE	Fluency A	VMT recall	VMT recognition
Surprise	0.185	0.082	0.209	0.074	-0.119	0.103
Disgust	0.346	0.019	0.072	0.125	0.396	0.090
Fear	-0.015	-0.481*	-0.103	-0.010	0.275	0.092
Anger	0.332	0.125	-0.057	0.338	-0.030	0.322
Sadness	0.587*	0.598**	0.134	0.262	0.077	0.302
Neutral	-0.110	-0.398	0.474*	0.439	0.523*	0.168
FERT total	0.565*	-0.010	0.285	0.542*	0.478*	0.554*

FERT: Facial Emotion Recognition Test; HADS: Hospital Anxiety and Depression scale; MMSE: Mini-Mental State Examination; VMT: Visual Memory Test of the Brief Cognitive Battery - happiness was not included because it was constant among all participants (all scored total); *p<0.05; **p<0.01.

**Figure 2.**
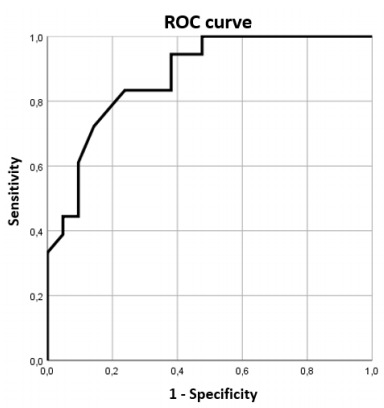
Receiver Operating Characteristic curve of predicted variability (AUC=0.877).

A binary logistic regression was performed in order to ascertain the variables significantly associated with stroke in a multivariate analysis. In the final model (step 4), the variables that remained significantly associated with stroke were: a higher score in the HADS-A, a lower score on VMT late recall, and sex (being male) (Table 3). The logistic regression model was significant [Hosmer-Lemeshow goodness of fit test (step 4): chi-square=3.930; p=0.788], and the predicted variability resulted in an area under the curve (AUC) of 0.877 in the ROC analysis (Figure 2).

**Table 3.** Final logistic regression model to predict stroke (step 4).

Predictive variable	B	SE	Wald	df	p-value	Odds Ratio	**95%CI for *Odds Ratio***	
							Lower	Upper
Sex	2.161	1.033	4.737	1	0.037	8.680	1.145	65.788
HADS A	0.394	0.150	6.940	1	0.008	1.483	1.106	1.989
VMT late recall	-1.153	0.450	6.570	1	0.010	0.316	0.131	0.762

B: B coefficient; SE: standard error; df: degrees of freedom; 95%CI: 95% confidence interval; HADS A: anxiety subscale; VMT: Visual Memory Test of the Brief Cognitive Battery.

ROC: Receiver Operating Characteristic.

## DISCUSSION

As far as we are concerned, this is the first study that investigated potential interactions between social cognition (i.e., facial emotion recognition) and post-stroke behavior (depression and anxiety) and cognitive (episodic memory and executive functions) symptoms. As previously reported,^15^ ^,^ ^16^ ^,^ ^17^ ^,^ ^29^ ^,^ ^30^ ^,^ ^31^ after an ischemic stroke, patients present with worse cognitive performance of immediate memory, late recall, recognition, and more depressive symptoms than healthy controls. There was no difference between stroke patients and healthy individuals in their ability to recognize facial emotion. However, the recognition of sadness expression positively correlated with the levels of anxiety and depression, while the recognition of fear expression negatively correlated with depression symptoms in the stroke group. Among cognitive variables, the recognition of neutral faces and FERT total score correlated positively with VMT recall, and FERT total score positively correlated with VMT recognition.

Contrary to our findings, Nijsse et al. reported significant differences between stroke patients and controls on FERT performance. Besides using a longer version of the FERT (60 *vs*. 25 figures in the current study), their patients had a stroke at least three years before the assessment.^32^ They also found that FERT scores correlated positively to behavioral problems reported by the Dysexecutive Questionnaire. Our results indicate that stroke, even with mild neurological impairment, is associated with memory deficits. More specifically, it confirms data on the emergence of post-stroke cognitive deficits without clear association with motor, sensory, or language deficits.^2^ Data from previous studies show that patients suffering from stroke have diminished ability to recognize facial, prosodic and lexical emotions in subacute and chronic phases, but the degree of neurological impairment was not considered (for a review, see Yuvaraj et al. The profile of mild neurological impairment in our patients may explain the absence of differences between patients in the subacute phase of the stroke and healthy individuals in their ability to recognize facial emotion.

Impairment in immediate memory, recall, and recognition was evidenced by the VMT, suggesting genuine memory deficits, not a change due to deficits in other cognitive domains, such as attention.^33^ Similar to our findings, Karimian et al. found that visual memory was the most impaired domain in patients in the chronic phase of the stroke as compared to healthy controls.^34^ Therefore, cognitive deficits start in the acute phase of stroke, remain in the subacute and chronic phases, and can progress to dementia.^1^ ^,^ ^2^ ^,^ ^3^ ^,^ ^4^ ^,^ ^6^ ^,^ ^7^ ^,^ ^10^

We found that stroke patients present high scores in HADS depression and anxiety subscales when compared to controls. Anxiety and depression are common in patients in the acute, subacute and chronic phases of the stroke.^30^ ^,^ ^31^ ^,^ ^35^ ^,^ ^36^ Actually, these are highly correlated constructs as shown here and by others.^5^ ^,^ ^6^ ^,^ ^29^ ^,^ ^37^ ^,^ ^38^ Among stroke patients, the higher the score in the HADS-A and HADS-DD, the worse the performance in the sadness and fear recognition of FERT, respectively. Our results are consistent with the literature showing that deficits in the ability to identify others’ facial emotions are associated with mood disorders.^39^ ^,^ ^40^

Beyond facial emotion recognition, other aspects, not evaluated in our study, such as theory of mind (TOM), social decision making and empathy are also important for social cognition.^32^ ^,^ ^41^ Even three to four years after a stroke, deficits in social cognition were found in patients, specifically in tasks of emotion recognition, TOM and of behavior regulation and inhibition.^32^ A more comprehensive assessment of social cognition, including other domains such as TOM, empathy, social decision making and behavior regulation, could have provided relevant information about deficits in the subacute phase of stroke, prompting earlier interventions.

In addition to anxiety, the score at the VMT late recall was significantly associated with stroke in the multivariate analysis. Greater decline in episodic memory was significantly associated to anxiety in a prospective study with healthy older individuals, suggesting that anxiety may be a predictor for cognitive decline.^42^ Among stroke patients with lesion in left hemisphere, lower episodic memory score was a significant predictor of higher anxiety scores one to three months after a stroke.^43^ Moreover, worse episodic verbal memory performance was a significant predictor of both higher anxiety and depression scores in patients three months after a stroke.^44^ These data indicate that anxiety is associated with episodic memory performance in the subacute phase of stroke. Studies with longitudinal design should confirm a causal link between them.

The results of the study must be interpreted in light of its limitations, including sample size and lack of neuroimaging assessment. In addition, it would have been of value to include patients with different levels, i.e. mild, moderate and severe, of neurological impairment. As shown by Circelli et al., changes in the prefrontal cortex, clinically observed through impaired performance on tasks of executive functioning, were associated with changes in visual scanning patterns for recognition of face emotions.^45^ Future research should better explore how neuropsychological performance and social cognition correlate, incorporating neuroimaging and neurophysiological information.

The current study supports the premise that post-stroke social cognition deficits should not be neglected, paving the way for future research with a focus on multidisciplinary prognosis and effective rehabilitation in order to achieve better recovery and favorable outcomes for these patients.
